# Nanomedicine, a valuable tool for skeletal muscle disorders: Challenges, promises, and limitations

**DOI:** 10.1002/wnan.1777

**Published:** 2022-01-29

**Authors:** Valentina Colapicchioni, Francesco Millozzi, Ornella Parolini, Daniela Palacios

**Affiliations:** ^1^ Italian National Research Council, Institute for Atmospheric Pollution Research (CNR‐IIA) Rome Italy; ^2^ Mhetra LLC Miami Florida USA; ^3^ Histology and Embryology Unit, DAHFMO Sapienza University Rome Italy; ^4^ IRCCS Santa Lucia Foundation Rome Italy; ^5^ Department of Life Sciences and Public Health Università Cattolica del Sacro Cuore Rome Italy; ^6^ IRCCS Fondazione Policlinico Universitario Agostino Gemelli IRCCS Rome Italy

**Keywords:** nanomedicine, scaffolds, skeletal muscle, target nanoparticles, tissue engineering

## Abstract

Muscular dystrophies are a group of rare genetic disorders characterized by progressive muscle weakness, which, in the most severe forms, leads to the patient's death due to cardiorespiratory problems. There is still no cure available for these diseases and significant effort is being placed into developing new strategies to either correct the genetic defect or to compensate muscle loss by stimulating skeletal muscle regeneration. However, the vast anatomical extension of the target tissue poses great challenges to these goals, highlighting the need for complementary strategies. Nanomedicine is an actively evolving field that merges nanotechnologies with biomedical and pharmaceutical sciences. It holds great potential in regenerative medicine, both in supporting tissue engineering and regeneration, and in optimizing drug and oligonucleotide delivery and gene therapy strategies. In this review, we will summarize the state‐of‐the‐art in the field of nanomedicine applied to skeletal muscle regeneration. We will discuss the recent work toward the development of nanopatterned scaffolds for tissue engineering, the efforts in the synthesis of organic and inorganic nanoparticles for gene therapy and drug delivery applications, as well as their use as immune modulators. Although nanomedicine holds great promise for muscle and other degenerative diseases, many challenges still need to be systematically addressed to assure a smooth transition from the bench to the bedside.

This article is categorized under:

Implantable Materials and Surgical Technologies > Nanotechnology in Tissue Repair and Replacement

## Graphical abstract

Nanomedicine offers potent therapeutic opportunities for skeletal muscle disorders, ranging from drug and gene delivery systems to immunomodulation and tissue engineering applications.

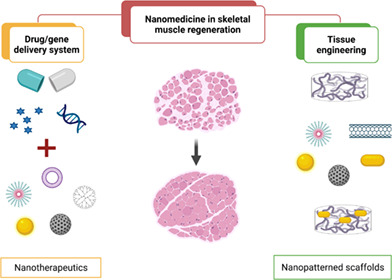

## 1 INTRODUCTION

Muscular dystrophies (MDs) comprise a group of rare genetic diseases that cause progressive weakness of skeletal muscle and the appearance of a dystrophic pathological phenotype. They are classified into nine major forms: myotonic, Duchenne, Becker, Limb‐girdle, facioscapulohumeral, congenital, oculopharyngeal, distal, and Emery–Dreifuss (Mercuri et al., 2019). Of them, the most prevalent form in adulthood is represented by myotonic dystrophies (DMs), affecting altogether 1 in 3000 people, and caused by mutations in the *DMPK* (DM1: # 160900) or *CNBP* (DM2: # 602668) loci (Mateos‐Aierdi et al., 2015). On the other hand, the most common and severe form of genetic dystrophy in childhood is Duchenne muscular dystrophy (DMD, ONIM: #310200), affecting around 1 in 5000 new‐born boys (Mendell et al., 2012) and caused by mutations in the dystrophin gene that result in the complete absence of the protein (Ervasti & Sonnemann, 2008; Hoffman et al., 1987). Overall, MDs involve mutations in over 40 genes that lead to different molecular mechanisms of pathogenesis (reviewed in (Mercuri et al., 2019). In addition to MDs, deficient muscle function is observed in the context of other physiopathological circumstances, such as extensive traumatic injury, atrophy due to cancer or muscle disuse (i.e., upon physical immobilization) (Sartori et al., 2021), or age‐related loss of muscle mass, sarcopenia (Muñoz‐Cánoves et al., 2020), representing a huge burden for the different National Health Systems. Therefore, strategies and interventions aimed at improving muscle function in both physiological and pathological situations remain a key challenge for the scientific and medical community.

In this context, nanomedicine offers a plethora of unprecedented tools that can revolutionize the way we look at regenerative medicine for skeletal muscle disorders. On the one hand, tissue regenerative nanomedicine employs nanoscale materials as drug delivery systems (DDSs) by exploiting the fact that endogenous transport at the cellular level is actively driven at nanometer length scale (Pozzi et al., 2014). The high surface‐to‐volume ratio of nanoparticles (NPs) facilitates the loading of growth factors (Z. Wang, Wang, et al., 2017), oligonucleotides (Roberts et al., 2020), cytokines (Raimondo & Mooney, 2018), and other bioactive agents to promote tissue regeneration while the abundant surface chemistry allows modifying the NPs with targeting ligands to assure a more precise delivery. By protecting their payload from degradation, NPs enhance their pharmacokinetics and bioavailability (Fathi‐Achachelouei et al., 2019). Regarding material composition, organic NPs (i.e., liposomes, polymers, solid lipid NPs) possess a long and successful clinical history and can assure good biocompatibility and biodegradability (Colapicchioni, 2020). Inorganic NPs (i.e., metal, oxides, carbon‐based, silica, etc.), on the other hand, show higher chemical stability, easier synthesis, and functionalization as well as good responsiveness to both internal (pH, temperature, redox potential) and external (light, ultrasound and magnetic field) stimuli (Mclaughlin et al., 2016). Furthermore, the unique optical properties (fluorescence, plasmonic absorbance, etc.) of these NPs permit their exploitation as imaging agents by allowing for superior spatiotemporal control within nanopatterned scaffolds or DDSs. However, despite these attractive properties, inorganic NPs are significantly less mature in terms of clinical translations and their potential toxicity is a significant matter of concern (Yang et al., 2019).

A second field in which nanomedicine has revolutionized skeletal muscle regeneration is in bioengineering approaches. A significant part of skeletal muscle regenerative studies is focused on synthesizing biomimetic scaffolds for cell attachment and growth to sustain tissue reconstruction. One of the major advantages of nanoscale materials is the possibility to optimize both the physical and biological properties of these scaffolds, allowing for highly tailored platforms. Different nanomaterials are exploited to optimize scaffolds' physical properties (i.e., mechanical strength, electroconductivity) and provide controlled bioactive agents release. In this context, nanofibrous scaffolds provide topographical support to guide myofiber differentiation and alignment by improving the system's architecture. On the other hand, electroconductive scaffolds exploit the intrinsic excitability of skeletal muscle tissue to modulate muscle cell survival, proliferation, and differentiation properties (Langridge et al., 2021).

**FIGURE 1:**
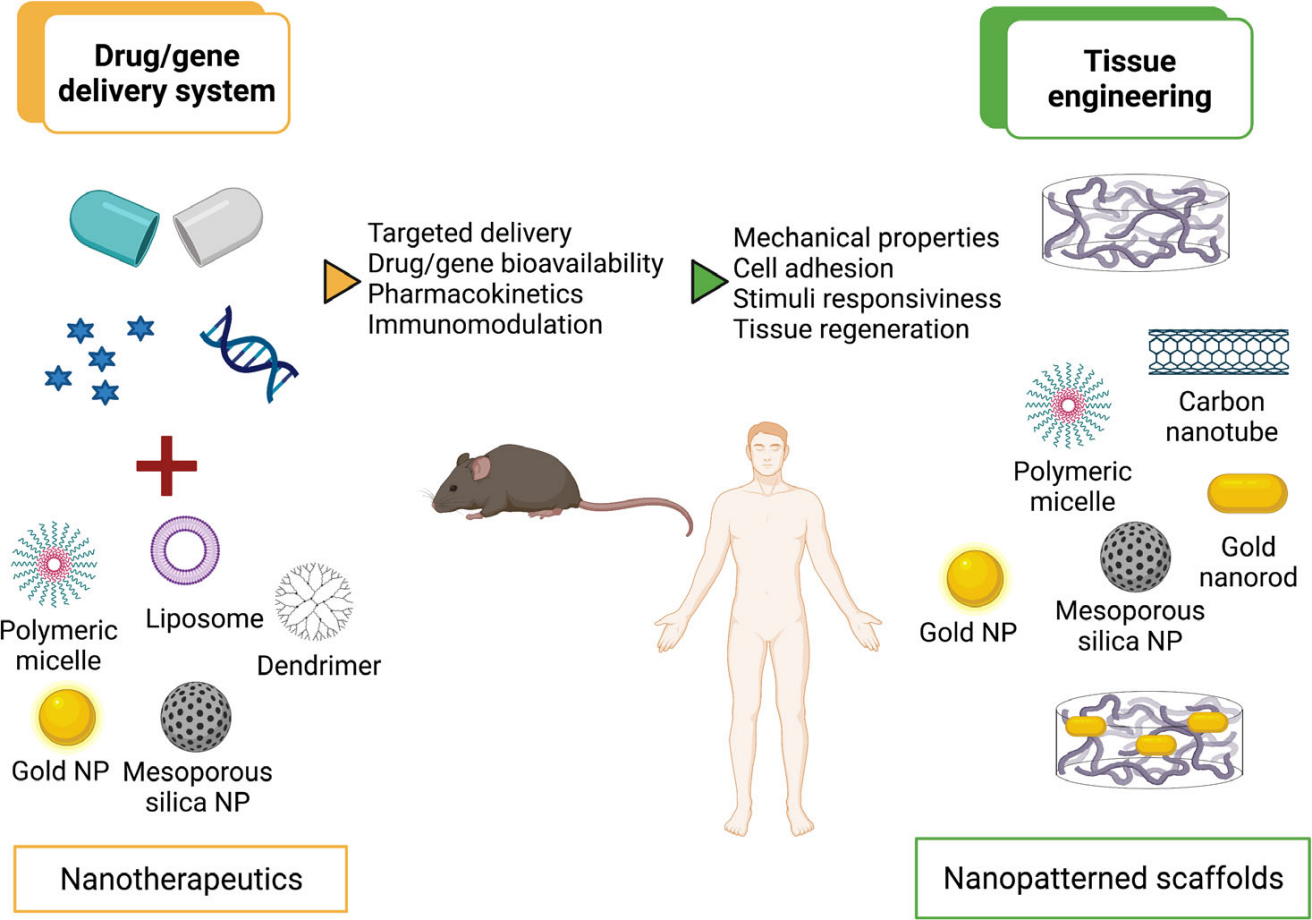
Nanomedicine in skeletal muscle regeneration. Overview of the different types of nanosystems used as drug delivery systems (left) or as nanopatterned scaffolds (right) for skeletal muscle regeneration

This review provides an overview of nanomaterials in muscle disorders focusing on their applications in tissue engineering approaches and as DDS, and exploring the intrinsic potential of some inorganic NPs to act as immunomodulators **(**Figure 1 **)**. This study will also discuss the future perspectives of this field and the difficulties limiting the efficient translation of these nanosystems from the bench to the bedside.

## 2 NANOPATTERNED SCAFFOLDS FOR SKELETAL MUSCLE TISSUE ENGINEERING

Although skeletal muscle tissue has a remarkable ability to regenerate upon injury, this ability is impaired after extensive traumatic damage, in congenital MDs, and during aging. Tissue engineering offers a promising alternative for tissue reconstruction when endogenous regeneration fails due to the extension of the lesion. Skeletal muscle can be engineered in vitro by using biodegradable and biocompatible scaffolds that favor myogenic cell adhesion, survival, proliferation, differentiation, and subsequent organization of muscle fibers prior to implantation in vivo. Different experimental models have been developed for skeletal muscle tissue engineering, ranging from in vitro models using different sources of cells, to in vivo transplantation into rodent models of skeletal muscle injury (i.e., volumetric loss models) (Khodabukus et al., 2018; Sicherer et al., 2020).

Tissue engineering combines anisotropic scaffolds, with myogenic cells and growth‐stimulating signals (referred to as the tissue engineer triad) to provide the structural support and external stimuli required to reconstruct muscle tissue (Chan & Leong, 2008). Although different approaches have been used for scaffold design, such as decellularized scaffolds (McCrary et al., 2020) and hydrogels (Lev & Seliktar, 2018), several issues need to be carefully considered and addressed, including biocompatibility and biodegradability, mechanical properties, architecture, bioactivity, and immunogenicity (Iravani & Varma, 2019; Langridge et al., 2021). For instance, even though decellularized extracellular matrix (ECM) scaffolds are the most physiologically relevant support for muscle growth, the availability of donor tissue, together with immunogenicity issues may limit their application in the clinics (McCrary et al., 2020). On the other hand, hydrogels, highly hydrophilic polymers of either natural or synthetic composition, are highly biodegradable and biocompatible but present poor mechanical strength and may need modifications to support cell adhesion, survival, and differentiation (Lev & Seliktar, 2018).

Ideally, the scaffold's material (i) should show excellent biocompatibility, (ii) have an optimized degradation rate (too slow degradation may render the cellular integration too restrictive while a too fast degradation may not provide sufficient protection), (iii) should actively interact with the cellular components to regulate their activities, and (iv) should also serve as a reservoir for exogenous growth factors. Furthermore, the scaffold should benefit from an interconnected pore structure and high porosity to guarantee cellular penetration. Finally, it should provide mechanical (i.e., by exerting traction forces) and shape stability to the tissue defect. Transferring these ideas into reality seem an daunting task.

Naturally derived polymers, such as collagen, chitosan, and gelatin alginate, have a good capacity to support cell attachment and show several advantages ranging from good biocompatibility to low‐cost production and availability (Alaribe et al., 2016). However, poor mechanical properties, rapid biodegradability, and immunogenicity are significant drawbacks that are limiting the in vivo application of natural polymers (Iravani & Varma, 2019). On the other hand, synthetic polymers (i.e., polycaprolactone [PCL], poly(L‐lactic acid) [PLA], polyglycolide [PGA], and their copolymer poly(lactic‐co‐glycolic acid) [PLGA]) can be easily tailored for specific applications, are often cheaper than decellularized scaffolds, have more controllable structure, and present less immunological issues than natural polymers. Although both PLA and PGA have been approved by the U.S. Food and Drug Administration (FDA), biocompatibility remains an open issue for many synthetic polymers. They also show poor efficiency in supporting cell attachment and poor affinity in physiological conditions (Alaribe et al., 2016).

**FIGURE 2:**
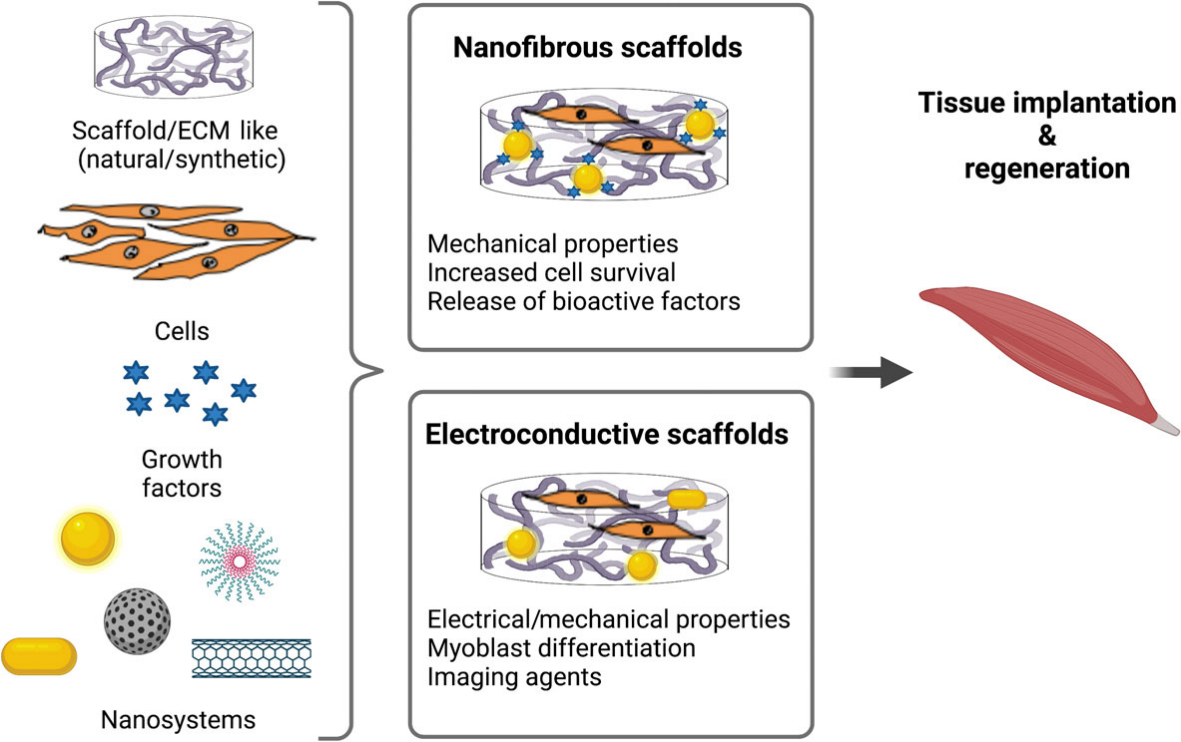
Nanopatterned scaffolds to reconstruct skeletal muscle. Traditional tissue engineering approaches combining scaffolds, cells, and growth factors can be implemented using a nanotechnology‐based strategy to modulate the electrical and mechanical properties of the scaffold, as well as to increase cell survival and differentiation prior to implantation. Two different types of nanopatterned scaffolds (nanofibrous and electroconductive scaffolds) have been developed for skeletal muscle regeneration

Despite the extensive work done in the past years, the inability of traditional engineered materials and scaffolds to finely control cellular functions, together with their poor biological, mechanical, and electrochemical properties, is significantly limiting tissue engineering's evolution and progress. In this context, the quickly evolving field of nanotechnology has the potential to solve, or at least mitigate, some of these obstacles. Nanopatterned scaffolds were first introduced in the early 2000s in an attempt to overcome the limitations of conventional scaffolds, and since then, they have established their merit in the bioengineering field (Evans et al., 2006). Nanofibrous and electroconductive scaffolds, in particular, are emerging as promising new tools in regenerative medicine **(**Figure 2 **)**.

### 2.1 Nanofibrous scaffolds

Nanofibrous scaffolds, defined as a mesh of nanoscale‐organized synthetic fibers (0–100 nm) that provide support for tissue regeneration, are showing great promise. They represent an accurate reproduction of the natural ECM, thus favoring cell adhesion, proliferation, migration, and differentiation (Pina et al., 2019). Compared to more traditional, solid‐walled scaffolds, nanofibrous scaffolds are more efficient in these functions due to their high surface‐to‐volume ratio (Gupta et al., 2014). They have been successfully used as substrates for many tissue engineering strategies, including reconstruction of bone, cartilage, nerve (Dong et al., 2020), and skeletal muscle (Patel et al., 2019). Different methods can be used to synthesize these nanofibrous polymer‐based scaffolds (i.e., thermally induced phase separation, self‐assembly, and electrospinning); however, since electrospinning allows to obtain anisotropic and geometrically aligned fibers, it is generally the preferred technique in skeletal muscle tissue engineering (Langridge et al., 2021). Electrospun polymers possess a wide array of physicochemical properties that can be modified by adding different molecules or by changing technical parameters (Y. Chen et al., 2020). As such, they have gained interest in tissue engineering approaches, as they provide the correct microenvironment for cell attachment, migration, proliferation, and differentiation (Pina et al., 2019).

As with traditional scaffolds, both naturally derived and artificial polymers have been used to generate nanofibrous scaffolds. Among the naturally derived polymers, Collagen I is widely used due to its mechanical stretch properties and its typical architecture made of small internal pores (Law et al., 2017; Parenteau‐Bareil et al., 2011). PCL has been deeply investigated among artificial polymers alone or in combination with other materials (Politi et al., 2020). An interesting work by L. Wang et al. (2015) have demonstrated that core‐shell scaffolds made of electrospun aligned nanofiber yarns (core) and photocurable hydrogels (shell), seeded with C2C12 myoblasts, enabled to induce the formation of three‐dimensional (3D), aligned, and elongated myotubes. Hydrogel nanofibers have also been widely investigated in tissue engineering (Lev & Seliktar, 2018) and have shown the capability to support the survival and maturation of myoblasts in both in vitro and in vivo studies (N. Rao et al., 2017).

Furthermore, due to their high surface area/volume ratio, nanofibrous scaffolds have also been explored as multitasking tools by incorporating nanocarriers loaded with bioactive agents (i.e., growth factors) to improve biofunctionality and stimulate tissue regeneration (Monteiro et al., 2015). NPs can be incorporated into the scaffold via advanced nanotechnology techniques. They play a crucial role in protecting the cargo from enzymatic degradation, enabling lower drug doses with decreased adverse side effects. Furthermore, NPs have the main advantage of providing a controlled release of bioactive molecules (Z. Wang, Wang, et al., 2017). Although this approach is still scarcely explored in skeletal muscle regeneration, promising results have emerged from applying PLGA NPs loaded with growth factors and incorporated in hydrogel scaffolds for brain (Y. Wang et al., 2013) and bone (Reyes et al., 2012) tissue regeneration. Furthermore, poly(ether)urethane‐polydimethylsiloxane/fibrin‐based scaffold containing PLGA NPs loaded with recombinant human vascular endothelial growth factor and basic fibroblast growth factor significantly promoted wound healing in a diabetes mouse model (Losi et al., 2013; Z. Wang, Wang, et al., 2017).

### 2.2 Electroconductive scaffolds

Electroconductive scaffolds have been developed to accommodate the important and intricate role of electrical signaling in native skeletal muscle tissues. They take into consideration evidence that muscle cell proliferation, adhesion, morphology, and maturation can be optimized by applying exogenous electrical stimulation. There are two main strategies to fabricate conductive scaffolds for tissue engineering: the use of conductive nanopolymers and the incorporation of conductive inorganic NPs into synthetic scaffolds (Nekounam et al., 2021).

Conductive polymers such as polyacetylene, poly(para‐phenylenevinylene), polyaniline (PANI), polythiophenes, and polypyrrole have received strong interest as conductive scaffolds for regenerative medicine (Nekounam et al., 2021). Due to their good biocompatibility and suitable biodegradability rate, polypyrrole and PANI have been combined both with PCL and hydrogels showing promising capabilities in enhancing myoblast differentiation and functional maturation (Langridge et al., 2021). For instance, conductive nanofibrous sheets composed of PANI blended with PLA were shown to enhance myogenic differentiation of H9C2 cardiomyoblasts (L. Wang, Wu, et al., 2017). A similar effect was obtained with a 3D nano/microfibrous scaffold mimicking electricals stimulation of skeletal muscle (Zhang et al., 2021). In addition, the incorporation of poly(3,4‐ethylene dioxythiophene) NPs to PCL scaffolds was shown to increase scaffold conductivity without affecting muscle cell viability (McKeon‐Fischer et al., 2015).

The second approach, based on the incorporation of conductive inorganic NPs, such as gold NPs, carbon nanotubes, and graphene (Jo et al., 2017; Saberi et al., 2019) into polymeric matrices, is facing more difficulties. Although the addition of carbon nanotubes to hydrogels improves both the mechanical and electrical properties of the scaffold and results in the enhancement of myoblast proliferation and maturation, the potential toxicity of these NPs represents a major problem (Langridge et al., 2021). Similarly, the nonbiodegradable nature of graphene significantly limits its application in tissue regeneration (Zor et al., 2019). In this context, the most promising strategy consists of incorporating gold NPs into scaffolds to make them electroconductive due to their low toxicity, good electroconductivity, and mechanical strength (Zor et al., 2019). For instance, the incorporation of polyelectrolyte‐coated gold nanorods has been shown to modulate cell‐mediated matrix remodeling of collagen scaffolds containing cardiac fibroblasts, revealing their potential use as antifibrotic agents (Sisco et al., 2008). Furthermore, the unique optical properties of gold NPs make them attractive imaging agents able to provide precise spatiotemporal monitoring of the regenerative process. Variable geometry allows fine‐tuning of their optical properties due to gold's plasmonic nature. In general, gold NPs exhibit better biocompatibility of other imaging agents such as quantum dots especially when they contain heavy metal ions such as Cd or Hg.

### 2.3 Cell sources for skeletal muscle tissue engineering

In addition to the scaffold composition and design, the source of muscle progenitors is a critical choice in tissue engineering approaches. In vivo muscle regeneration is a multistep process that begins with the activation of a population of quiescent muscle‐resident stem cells called satellite cells. Upon injury, activated satellite cells start to proliferate, expand as committed myoblasts, and migrate to the site of the lesion, where they exit the cell cycle and terminally differentiate into multinucleated myofibers (Brancaccio & Palacios, 2015). Satellite cell function is highly influenced by the activity of other muscle‐resident cells, such as immune cells and fibro‐adipogenic progenitors, that act via paracrine signaling (Dumont et al., 2015). The regenerated muscle's full functionality is achieved when the newly formed myofibers are innervated by neural cells and the neo‐regenerated tissue is vascularized. Given the complexity of the process, it is not surprising that engineering skeletal muscle in vitro has posed many challenges.

For the source of myogenic precursors, different types of cells have been explored. The first studies used C2C12 immortalized murine myoblasts, as they can be easily expanded and differentiated in vitro. Mouse (Carosio et al., 2013) or rat (Corona et al., 2014; Y.‐C. Huang et al., 2005; Machingal et al., 2011) muscle progenitors have also been extensively used. However, although these experiments were crucial for developing and optimizing different protocols for skeletal muscle bioengineering, rodent cells are not suitable for clinical applications due to immune‐compatibility issues. Another possibility is to use human myogenic progenitors, isolated from skeletal muscle biopsies (Madden et al., 2015; Powell et al., 1999), human mesangioblasts (Fuoco et al., 2015) or human embryonic stem cells (hESCs). However, the major drawbacks of these approaches are the scarcity of available cells, the need to use immune‐compatible donor cells, and ethical concerns in the case of hESCs. A considerable advance in the field came from developing induced pluripotent stem cell (iPSCs) technology. Human iPSCs have been successfully used in skeletal muscle tissue engineering studies (Maffioletti et al., 2018; L. Rao et al., 2018) and to study the physiopathological characteristics of different MDs, by using patient‐derived iPSCs (Maffioletti et al., 2018).

In addition to the source of myogenic precursors, co‐culture with other muscle‐resident populations such as endothelial or neural cells should improve the final outcome of the biomimetic tissue. In this sense, pioneer work from the Tedesco lab showed that multilineage differentiation of human iPSCs can be used to obtain the different muscle‐resident cell types that can then be co‐cultured using fibrin hydrogels. Using this approach, the authors provided proof‐of‐principle of the in vitro generation of complex, 3D, multilineage, artificial skeletal muscles from human iPSCs (Maffioletti et al., 2018).

Finally, as discussed above, vascularization of the artificial tissue is essential to provide both nutrients and oxygen. However, this is a technically complex process that can be improved through microfluidics tissue engineering methods, such as soft lithography, rapid prototyping, and bioprinting (Wan et al., 2020). Optimization of these protocols for future clinical applications, however, remains a critical issue.

### 2.4 Microfluidics for tissue engineering

Despite their potential, nanopatterned scaffolds present a great degree of complexity in their synthesis and management by clinicians (Iravani & Varma, 2019). In this context, microfluidic‐based techniques, such as soft lithography or 3D bioprinting, have emerged as appealing technologies for reconstructing skeletal muscle tissues in an automated and controlled manner.

Soft lithography is the primary method used to build micropatterned substrates and it is now finding application in nanopatterned scaffolds for skeletal muscle tissue engineering (Jana et al., 2016). The term comprises a series of techniques in which a soft elastomeric stamp is used for printing patterns of different sizes, shapes, and materials (Lindquist et al., 2012). Compared to other modeling techniques, its advantages include a simple configuration accompanied by elevated productivity, a relatively low cost, and a wide resolution, ranging from nanometers to micrometers. (Sahin et al., 2018). Although these techniques have not been widely applied yet to skeletal muscle engineering, promising results have been obtained (Bian et al., 2009; H. Gao et al., 2021; Gattazzo et al., 2018; Hwang et al., 2017; Wan et al., 2020). One of the pioneering works in the field used high aspect ratio soft lithography to build polydimethylsiloxane (PDMS) molds containing arrays of mesoscopic posts (Bian et al., 2009). When used to fabricate fibrin hydrogels containing muscle cells, these posts enhanced nutrient diffusion and favored local 3D cell alignment by governing the spatial pattern of mechanical tension (Bian et al., 2009). More recently, Bonaldo's group also used soft lithography to drive the alignment and differentiation of C2C12 myoblasts in gelatin‐genipin based hydrogels. Interestingly, their acellular scaffolds were shown to be highly biocompatibile and displayed a slow biodegradation rate after in vivo implantation in mouse muscles, suggesting they could represent useful biomaterials for skeletal muscle engineering (Gattazzo et al., 2018). A different approach, based on the combination of soft lithography and melt‐casting was used by H. Gao et al. (2021), to build PLGA micro‐grooved substrates to study the cellular response of C2C12 myoblasts. Their results showed, once again, the important role of substrate in modulating proliferation, differentiation, and alignment.

While soft lithography is usually used to fabricate planar structures, a major challenge in designing 3D functional artificial tissues is achieving correct nutrient and oxygen delivery, especially in thick tissues such as skeletal muscles. To overcome this limitation, Wan et al. (2020) developed a thermo‐responsive polymer and micro‐milling method for building vasculature into 3D synthetic muscles. Advanced microfluidics techniques, such as rapid prototyping and bioprinting, also have the potential of building biomimetic 3D structures, and have been explored in different areas of tissue engineering, including skeletal muscle regeneration (Ostrovidov et al., 2019). 3D bioprinting can be used to fabricate 3D structures made of natural or artificial polymers (i.e., collagen, fibrin, nanofibers) and containing cells and bioactive molecules in a predesigned structure (Song et al., 2021; Zhuang et al., 2020). It has several advantages compared to other tissue engineering strategies. First, the possibility of obtaining anisotropic structures is essential for proper cell alignment and maturation (Capel et al., 2019; Zhuang et al., 2020). In addition, the most commonly used biomaterial PDMS presents high biocompatibility and is highly permeable to gas, allowing proper oxygen delivery. This is particularly important in the construction of 3D complex tissues, such as skeletal muscle (Reid et al., 2020).

The two most commonly used techniques used include inkjet printing and extrusion‐based printing (Sorkio et al., 2018). Inkjet printing is based on a drop‐by‐drop bioink deposition (Gudapati et al., 2016) Extrusion‐based printing relies on pushing the bioink through a nozzle using pneumatic or mechanical pressure (Ostrovidov et al., 2019). Extrusion‐based bioprinting allows the use of bioinks with a wide range of viscosities, which results in high viability (over 80%), and it is relatively fast (<0.05 mm/s) (Jian et al., 2018). Both techniques have limitations; while inkjet printing has a resolution problem (Costantini et al., 2017), extrusion‐based printing, by exerting shear forces, can damage cells and modify both the mechanical properties and architecture of the constructs, with a significant negative impact on their performance and effectiveness (Langridge et al., 2021).

Hybrid strategies have been implemented to overcome these limitations. Costantini et al. have introduced an innovative hybrid 3D bioprinting approach based on a microfluidic printing head coupled to a co‐axial needle extruder for high‐resolution 3D bioprinting of polyethylene glycol (PEG)/fibrinogen hydrogel fibers laden with C2C12 muscle cells. When implanted in immunocompromised mice, these bioprinted constructs, containing a functional morphology, can generate organized artificial muscle tissue (Costantini et al., 2017). On the other hand, Yeo et al. (2016) have combined extrusion‐based bioprinting with electrospinning to create a 3D structural shape containing aligned nanofibers (obtained with optimized electrospinning) and cell‐printed myoblasts. 3D bioprinting has also been recently used to successfully create skeletal muscle scaffolds containing neural cells. This approach restores muscle function in a rat model of muscle injury (Kim et al., 2020).

In summary, 3D bioprinting reveals a promising strategy to reconstruct artificial skeletal muscles for research and (future) clinical applications.

## 3 NANOCARRIERS AS DRUG AND GENE DELIVERY SYSTEMS IN MUSCLE DISORDERS

### 3.1 NP‐mediated drug and gene delivery

An alternative approach to tissue engineering that is gaining interest mainly for the treatment of genetic muscle disorders is based on the stimulation of endogenous regeneration. This can be done through either cellular, pharmacological, or genetic approaches. Among the different therapeutic agents, anti‐inflammatory and pro‐regenerative drugs, oligonucleotides, and plasmids can be used to palliate the disease symptoms, correct the genetic defect or stimulate the regeneration potential of diseased muscles. However, gene and drug delivery into skeletal muscle tissue present several caveats, including bioavailability, toxicity, and the vastness of the target tissue. Therefore, the development of strategies to improve delivery of therapeutically relevant doses of the molecules while reducing off‐target effects is an urgent issue. In this sense, much attention has been recently put into nanotechnology and its potential to develop new bio carriers for muscle diseases that overcome some of these problems.

One of the major advantages of using nanosystems as biocarriers for muscle disorders is the possibility to optimize their biophysical and biological properties, which is fundamental when considering them as DDSs. From a chemical point of view, the current nanosynthetic methods allow to synthesize NPs as highly reproducible populations of various sizes and shapes (Boselli et al., 2020). It is well recognized that size is a crucial factor being directly related to both circulation time and the fate of nanocarriers, for example, NPs smaller than 10 nm are rapidly removed from blood circulation in the kidneys (Cole & Holland, 2015). Regarding their charge, it has been observed that cationic NPs, regardless of composition, are often associated with toxic effects (McConnell et al., 2016). Finally, despite “nano‐flowers” and “nano‐stars” being interesting systems, especially in terms of their biological identity (Boselli et al., 2020), the most commonly used shapes in nanocarriers are rods and spheres.

NP‐mediated drug delivery has been proposed as a promising alternative to allow therapeutic doses of drugs in the target tissue while reducing toxicity. Many efforts are now being placed to design nanostructures for drug delivery into specific tissues. However, the use of nanostructures as therapeutic biocarriers in skeletal muscles has not been extensively applied yet, and research is still mainly limited to cellular models. Interestingly, the first proofs‐of‐principle in animal models are now emerging and showing promising results **(**Table 1 **)**. Among the different nanostructures used as DDS into cells of the skeletal muscle lineage, particular emphasis has been put on some inorganic NPs, such as silica and metal NPs, and into polymeric nanostructures, such as PLGA, due to their high loading capability and good biocompatibility.

**TABLE 1:** Summary of nanoparticles used as DDS into skeletal muscle in vivo

Type of nanoparticle	Size	Drug/oligonucleotide	Application	Animal model	References
*Inorganic*					
PEGylated gold NPs	∼100 nm	IL‐4	Macrophage polarization	Ischemic injury C57BL6/J mice	Raimondo and Mooney (2018)
Perfluorocarbon NPs	160–240 nm	Rapamycin	Modulation of autophagy	Mdx mice	Bibee et al. (2014)
Gold NPs	∼15 nm	CRISPR RNP complexes, pDNA	Gene editing through HDR Dystrophin recovery	Mdx mice	Lee et al. (2017)
*Organic*					
PEGylated nanoliposomes	~80 nm	Methylprednisolone	Anti‐inflammatory	Mdx mice	Turjeman et al. (2019)
Hybrid liposomes	60–90 nm	Gentamycin	Read‐through dystrophin recovery	Mdx mice	Yukihara et al. (2011)
PLA NPs	∼200 nm	Rapamycin	Immunomodulation	AAV‐infected C57BL/6 mice Cynomolgus monkeys	Meliani et al. (2018)
PLGA‐PEG	∼100 nm	PTEN inhibitor	Improvement of muscle function	Mdx mice	D. Huang et al. (2020)
PEAs	<200 nm	pDNA	Proof‐of‐principle of gene delivery	Mdx mice	M. Wang, Tucker, et al. (2012)
PPE‐EA		b‐Galactosidase pDNA	Proof‐of‐principle of gene delivery	Balb/c mice	J. Wang et al. (2002)
Polyplex nanomicelle	∼100 nm	Luciferase pDNA sFlt‐1 pDNA	Proof‐of‐principle of gene delivery	Balb/c mice	Itaka et al. (2010)
tcDNA	40–100 nm	ASOs	Dystrophin recovery	Mdx mice	Goyenvalle et al. (2015)
PEG‐PEI polyplexes		ASOs	Dystrophin recovery	Mdx mice	Williams et al. (2008)
PLGA‐encapsulated PEG‐PEI‐ASOs	215–240 nm	ASOs	Dystrophin recovery	Mdx mice	Sirsi et al. (2009)
ZM2 NPs	∼137 nm	ASOs	Dystrophin recovery	Mdx mice	Ferlini et al. (2010), Falzarano et al. (2014)
Nanocapsule	25 nm	CRISPR RNP complexes	Somatic gene editing	Ai14 mice	G. Chen et al. (2019)
PPA NPs	∼15 nm	Cy5.5‐label	Proof‐of‐principle of targeted delivery	Ischemic injury Sprague Dawley rats	Ungerleider et al. (2017)

Abbreviations: AAV, adeno‐associated virus; ASOs, antisense oligonuclecotides; HDR, homologous DNA repair; NP, nanoparticle; pDNA: plasmid DNA; PEA, polyester amine; PEG, polyethylene glycol; PEI, polyethyleneimine; PLA, poly(lactic acid); PLGA, poly(lactic‐co‐glycolic acid); PPA, peptide polymer amphiphiles; PPE‐EA, poly(2‐aminoethyl propylene phosphate); RNP, ribonucleoprotein; tcDNA, tricycloDNA; ZM2, cationic core‐shell NPs made of a polymethyl methacrylate (PMMA) core and a copolymer shell consisting of units derived from *N*‐isopropyl‐acrylamide+ (NIPAM).

For instance, mesoporous silica NPs have been used to deliver secretase inhibitors into muscle cells, modulating the activity of the Notch pathway and inducing the differentiation program (Böcking et al., 2014). Due to their versatility, these NPs have attracted much attention in nanomedicine (Colapicchioni et al., 2015) and they are expected to address certain limitations to both drug delivery and tissue engineering (Rosenholm et al., 2016). Mesoporous silica NPs can be considered as intermediary carriers: they show similar biocompatibility as organic NPs and the durability and versatility of inorganic nanovectors. They offer a set of unique and outstanding structural properties, such as high surface area (>1000 m^2^/g), pore volume (>1.0 cm^3^/g), stable mesostructure, and modifiable morphology that meets the need of regenerative medicine, making them appealing both as DDS and in nanopatterned scaffolds (Rosenholm et al., 2016). When considered as DDSs, the large surface area of these NPs and their pore volume allow for high loading, while the tunable diffusional drug release from the mesoporous structure gives rise to a biogenic local concentration at the targeted area. However, in a comparative study using different types of NPs, Guglielmi et al. (2019) showed that at high doses and long incubation times, mesoporous silica NPs might lead to toxicity of primary human myoblasts, as compared to polymer‐based nanostructures such as PLGA NPs. The FDA has approved PLGA NPs for clinical use in humans as DDSs. Along these lines, they have been exploited both in cancer and Alzheimer's disease nanomedicine and they are particularly attractive also in tissue remodeling (Danhier et al., 2012). These polymeric NPs have been used for growth factor loading and delivery to promote neovascularization and tissue regeneration (Jin et al., 2017). PLGA is a copolymer composed of PLA and PGA and its NPs can be synthesized by using various protocols, including phase separation, emulsion solvent diffusion. However, emulsification–solvent evaporation is generally the most used method. This technique allows encapsulating hydrophobic drugs while its variation, known as double or multiple emulsion, makes it possible to entrap hydrophilic molecules (Z. Wang, Wang, et al., 2017). PLGA NPs are biodegradable and in the biological environment, they undergo hydrolysis to produce the original monomers and the degradation products are easily metabolized via the Krebs cycle (Anderson & Shive, 2012).

Finally, gold NPs have been shown to efficiently deliver drugs, oligonucleotides, and plasmids into skeletal muscle cells. Gold NPs are emerging as versatile DDS due to their enhanced cellular uptake, flexibility in functionalization, and the FDA has approved them for medical applications. They can be easily tuned to various sizes and shapes, including spheres, rods, cages, and shells. On a general note, gold NPs have been widely explored in drug delivery and tumor thermal ablation, they have been studied and administered in Phases I and II clinical trials for cancer treatments (refer to https://clinicaltrials.gov/show/NCT02761525 NLM) (Urie et al., 2018).

Despite their enormous potential as DDS, currently, only a few NPs have been tested yet in preclinical mouse models of muscle disease for either drugs, oligonucleotides, or gene delivery (Table 1). Regarding drug delivery using NPs in vivo, gentamycin‐loaded hybrid liposomes composed of l‐α‐dimyristoylphosphatidylcholine, and polyoxyethylene (23) lauryl ether (C₁₂(EO)₂₃) have been shown to increase the number of dystrophin‐positive muscle fibers in a mouse model of DMD (Yukihara et al., 2011). Aminoglycoside antibiotics, such as gentamycin, act through a read‐through mechanism, allowing ribosomes to continue protein synthesis through premature termination codons (Howard et al., 1996). As nonsense mutations affect 15% of DMD patients, these antibiotics provide an interesting therapeutic opportunity. In this sense, the use of NPs as DDS can reduce toxicity and increase the bioavailability of the therapeutic molecule.

On the other hand, rapamycin‐containing inorganic NPs, based on perfluorocarbon (PFC), have been recently used to modulate autophagy in the same DMD mouse model (Bibee et al., 2014). From a therapeutic perspective, autophagy was recently shown to be a druggable target to improve skeletal muscle regeneration (Fiacco et al., 2016). Interestingly, as compared to oral preparations, intravenous delivery of rapamycin‐loaded PFC NPs further improved muscle function in a mouse model of DMD and modulated the autophagic response (Bibee et al., 2014). In addition, rapamycin is an immune‐suppressing drug (Thomson et al., 2009) and rapamycin‐loaded PLA NPs have been recently used as immunomodulators in adeno‐associated virus (AAV)‐mediated gene therapy approaches, allowing vector re‐administration (Meliani et al., 2018). The use of different NPs as modulators of the immune response is now emerging as a promising tool in regenerative medicine and muscle disorders. It will be further discussed in a separate section.

Before moving on, and as a word of caution, it is worth mentioning that increasing evidence shows that internalization itself may modulate skeletal muscle cell function for some type of nanostructures. For instance, the internalization of silica NPs has been shown to promote skeletal muscle cells fusion in vitro, although the molecular mechanisms by which this occurs are not fully understood yet (Poussard et al., 2015). On the other hand, gold and gold–silver NPs internalization enhances myogenic differentiation and promote skeletal muscle regeneration through a mechanism involving the p38α kinase pathway (Ge et al., 2018), a key signaling pathway in the regulation of skeletal muscle function that is altered in physiological and pathological conditions, such as MDs or aging (Brancaccio & Palacios, 2015). These observations should be taken into account when using NPs as DDSs in muscle disorders.

### 3.2 Nanocarriers for gene therapy

#### 3.2.1 NPs for gene delivery

**FIGURE 3:**
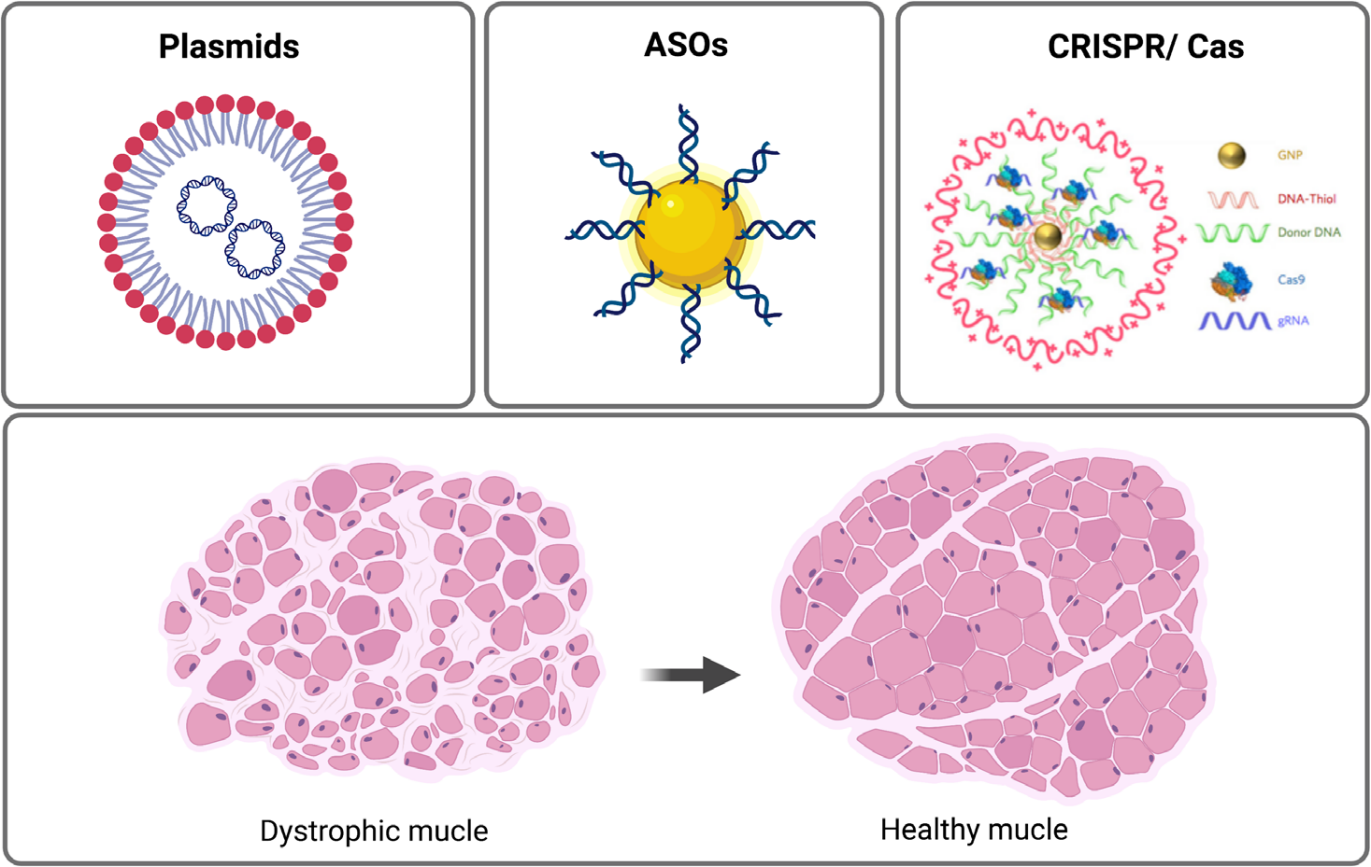
Nanomedicine‐based approaches for gene therapy applications in skeletal muscle disorders. Different types of nanoscale structures have been developed to introduce plasmids (left panel), oligonucleotides such as anti‐sense oligonuclecotides (ASOs, middle panel), and the components of the CRISPR/ Cas gene editing machinery (right panel, reprinted with permission from Lee et al., 2017) into skeletal muscle cells and tissues. The overall goal is to obtain a functional protein to recover muscle functionality

The treatment of rare genetic diseases, such as MDs, remains one of the major challenges in medical practice. Different strategies are being pursued to recover a functional copy of the damaged gene. The first is based on delivering a functional copy of the defective gene, which can be achieved through either cell transplantation or via the delivery of plasmids into dystrophic muscles. Whereas cell therapy for muscle disorders present several caveats regarding safety and efficacy issues and will not be discussed here (Judson & Rossi, 2020), gene therapy is challenged by the high molecular weight of therapeutic plasmids, enzymatic degradation and the anionic nature of nucleic acids (Elsabahy et al., 2011; Silva et al., 2015). Currently, there are only three FDA and/ or the European Medicines Agency (EMA) approved Phase I or I/II clinical trials aimed at assessing the safety, biological activity, and efficacy in MDs, all of them delivering a shorter version of dystrophin (micro‐ or mini‐dystrophin) in a small cohort of DMD boys (Duan, 2018; Verhaart & Aartsma‐Rus, 2019) and one approved treatment for spinal muscular atrophy (SMA), a neuromuscular disease (Shahryari et al., 2019). All of them are based on the use of nonreplicating recombinant AAVs to deliver the gene, which presents several clinical drawbacks. Despite being relatively safe in small laboratory animals, several studies have shown the potential toxicity of high dose systemic AAV‐delivery in large animals (Duan, 2018). Moreover, a significant proportion of the population has preexisting resistance to AAV, starting early in childhood (Li et al., 2012), which makes them ineligible for the treatment. Even for those patients who do not present resistance, the treatment itself activates a strong adaptive immune response, impairing second dosing if needed. Therefore, despite AAVs currently being the main delivery system for gene therapy in skeletal muscle tissue, and several AAV‐derivatives being in the advanced phase of clinical development in gene therapy applications for muscle disorders (Duan, 2018) they are not exempt from concerns. Among them, their relatively low packaging capacity, the lack of tissue selectivity and inefficient targeting of muscle stem cells, and the elicitation of a strong immune response. Therefore, the urge to explore alternative delivery methods has opened the door to nanotechnology as a complementary tool to deliver genes into skeletal muscles (Nance et al., 2018). Although still far from the clinical setting, several attempts to introduce plasmids into muscle cells and tissues using polymeric nanostructures have been developed **(**Figure 3 **)**. For instance, in a recent work, Jativa et al. (2019) used a 5‐polyamidoamine dendrimer (G5‐PAMAM) conjugated to a muscle homing peptide to introduce a luciferase plasmid into C2C12 cells. In addition, hyper‐branched poly(ester amine)s (PEAs) have been successfully used to transfect plasmids into C2C12 cells and dystrophic muscles with low toxicity, likely due to the fact that PEAs are highly biodegradable (M. Wang, Tucker, et al., 2012). In a different study, sustained release of a β‐galactosidase plasmid into mouse muscles was obtained via intra‐muscle injection of biodegradable polyphosphoester, poly(2‐aminoethyl propylene phosphate) (PPE‐EA) complexes (J. Wang et al., 2002). Finally, polyplex nanomicelles also showed sustained transgene expression in skeletal muscle when introduced systemically via intravenous injection (Itaka et al., 2010). However, the difficulties of efficiently delivering plasmids into such an extended tissue as skeletal muscle are evident and, to date, none of the nanotechnology‐based approaches has allowed efficient transfection of the therapeutically relevant amount of plasmids in living animals.

A different approach to gene delivery would be the delivery of mRNAs encoding a functional version of the damaged gene. However, technical limitations to RNA delivery in vivo have for a long time tampered the use of mRNAs as therapeutic molecules (Servick, 2020). This has been in part recently overcome by the enormous success of the COVID‐19 mRNA vaccines developed by Pfizer/BioNTech and Moderna, which have demonstrated the potential of lipid NPs to deliver mRNA in vivo. If this strategy will reveal successful for treating muscle disorders is still unknown. Among the challenges, as discussed for plasmid delivery, the difficulty to specifically target skeletal muscle tissue, and the need to avoid undesired, off‐targets effects.

#### 3.2.2 NPs for oligonucleotide delivery

Technically easier than gene delivery, a different strategy to modulate gene function in muscle disorders is based on the delivery of oligonucleotides, such as anti‐sense oligonuclecotides (ASOs), small interference RNAs (siRNAs), and microRNAs (Hammond et al., 2021). At the time of this review, 13 oligonucleotide‐based drugs have been approved by the FDA and/or the EMA for their use in humans. ASOs are short single‐stranded DNA or RNA oligonucleotides that recognize complementary regions at target genes or transcripts through Watson–Crick base pairing. They exert their action through different molecular mechanisms, such as RNase‐H1‐mediated degradation of target mRNAs, microRNA inhibition or regulation of the splicing machinery and show high potential as therapeutic agents for genetic diseases (Crooke et al., 2021). In particular, for neuromuscular disorders, ASOs to treat SMA and DMD are already available in the clinics and several others are in advance phases of investigation (Crooke et al., 2021; Hammond et al., 2021). However, in vivo delivery of ASOs into skeletal muscle tissue present several challenges, including the vast extension of the target tissue, the instability of these molecules due to the presence of endogenous nucleases and relatively low affinity. Some of these issues, like stability or affinity for target nucleic acids, have been addressed through chemical modifications of the oligonucleotide, such as backbone modifications, including phosphorothioate (PS) linkages or phosphorodiamidate morpholino oligonucleotides (PMOs), and sugar modifications, like methylation of the 2′ residue (2′O_Me) (Roberts et al., 2020). However, there is still a need to improve ASO chemistry and to develop safer and more efficient biocarriers for oligonucleotide delivery into target skeletal muscles.

Within this context, several approaches based on nanostructures are being actively explored in cellular models and preclinical settings. In a recent work, 2′O_Me/PS‐modified ASOs (which are negatively charged), complexed to two different cationic cell‐penetrating peptides, nona‐arginine and PepFect14, were delivered into human myoblasts derived from patients affected of MD type 1 (DM1) (van der Bent et al., 2019). In DM1 patients, a large trinucleotide expansion within the *DMPK g*ene leads to an aberrant transcript and RNA‐mediated toxicity. In their work, van de Bent et al. (2019) showed that 2′O_Me/PS‐modified ASOs complexed to cationic cell‐penetrating peptides efficiently arrive to the nucleus of DM1 patient‐derived cells, where they reduce the formation of nuclear aggregates, a hallmark of DM1 disease. Another mechanism of action of ASOs is through exon skipping in patients containing frame‐shift mutations of the defective gene. In this case, ASOs act by modulating the splicing machinery so that the target exon(s) are included or excluded from the mature transcript, to either increase the levels of the protein or to obtain shorter but still functional protein (Crooke et al., 2021). ASOs have been proved efficient in treating several neuromuscular disorders, such as SMA and DMD. In DMD patients three PMO ASOs (eteplirsen, golodirsen, and vitolarsen) are now approved as therapeutic molecules for DMD patients with eligible mutations (Aartsma‐Rus & Corey, 2020; Duan et al., 2021). Despite their clear benefits, some issues, such as insufficient protein recovery or inefficient targeting of the heart, leave the door open to further improvement of the technology, which is now focused on improving systemic delivery. In this direction, Goyenvalle et al. (2015) recently showed that ASOs made of tricycloDNA (tcDNA_ASOs), which spontaneously form NPs of 40–100 nm, can efficiently target skeletal muscles, heart and brain, promoting a high recovery of a partially functioning dystrophin in DMD mice. Moreover, work from Lutz's lab demonstrated that different nanopolymers facilitate ASO delivery and exon skipping in mdx mice (Sirsi et al., 2009; Williams et al., 2008). Finally, Ferlini et al. (2010) used polymethyl methacrylate PMMA/N‐isopropil‐acrylamide (ZM2) NPs as ASOs biocarriers into skeletal muscle in vivo. Altogether, these examples suggest that conjugation of ASOs to different nanomaterials can improve their bioavailability, tissue targeting, subcellular localization and therapeutic action.

Despite the aforementioned progress, studies focused on ASOs also revealed many caveats, as expected for any emerging technology. For instance, recent work from Ferlini's lab studied chitosan‐shelled nanobubbles as a DDS for PMO ASO to suppress the expression of Double homeobox 4 *(DUX4)* in a facioscapulohumeral muscular dystrophy (FSHD) cell model (Falzarano et al., 2021). *DUX4,* which is not normally expressed in muscle cells, is associated with cell toxicity and the deregulation of several downstream genes (J. C. Chen et al., 2016; Yao et al., 2014). DUX4‐induced gene expression is the major molecular signature in FSHD skeletal muscle (Yao et al., 2014). Consequently, several emerging therapeutic approaches aim at silencing *DUX4* in affected cells. The choice of chitosan as DDS aligns with the latest trends in nanotechnology. Indeed, this linear polymer of glucosamine/acetylglucosamine obtained from crustacean shells has gained more and more attention due to its biocompatibility (it has also received the regulatory approval), biodegradability and green synthesis (Lombardo et al., 2020; Sheir et al., 2021). The encapsulation testing done by Falzanaro and co‐workers confirmed the good loading capability of the chitosan NPs; however, the experiments in cell cultures showed a lack of *DUX4* gene silencing, due to irreversible loading of the ASO in chitosan nanobubbles (Falzarano et al., 2021).

siRNAs represent an alternative to ASOs for silencing gene expression. These double‐stranded RNAs are composed of a guide strand complementary to the target mRNA and a passenger strand, They act through the endogenous RNA‐induced silencing complex to induce degradation of the target mRNA (Hammond et al., 2021; Watts & Corey, 2012). siRNAs currently represent the gold‐standard for gene silencing in in vitro studies and have already entered the clinics as therapeutic molecules. To date there are three marketed products based on siRNAs (none of them for muscle disorders), and several more are under development. However, as with ASOs, clinical applications in MDs are still limited by their degradation by serum nucleases, lack of targeted delivery and poor tissue uptake (Hammond et al., 2021).

More work is still needed to elucidate the contribution of nanotechnologies to delivery of oligonucleotides (ASOs, siRNAs, microRNAs, etc.) to skeletal muscle tissue in the treatment of MDs.

#### 3.2.3 NPs for gene editing

Last, another field in which nanotechnologies show an enormous potential is as biocarriers within the context of gene editing strategies, and in particular in those involving the Nobel prize awarded CRISPR/Cas technology (Doudna & Charpentier, 2014). Based on an adaptive immune response against viruses in prokaryotes, CRISPR/Cas gene editing requires a Cas nuclease and two RNA strands, termed CRISPR RNA (crRNA) and trans‐activating RNA (tracrRNA), which can be artificially fused into a chimeric structure called single guide RNA (sgRNA). Inside the cell, the Cas/gsRNA ribonucleoprotein (RNP) complex recognizes the target sequence and induces a double‐strand break (DSB). Once the DSB has occurred, the cell use one of two possible mechanisms to repair the damage: nonhomologous end joining (NHEJ) and homologous DNA repair (HDR). The latter of which, requires proliferating cells to function, allows the introduction of point mutations with high selectivity in the presence of a donor DNA. This approach is, at least in theory, the preferred pathway for correcting genetic mutations (Hsu et al., 2014).

Elegant studies in model animals have recently shown the feasibility of CRISPR/Cas‐based gene editing in muscle disorders. Seminal work from the Olson, Gersbach, and Wagers laboratories independently demonstrated CRISPR/Cas can be used to recover a partially functioning dystrophin via a NHEJ mechanism in a mouse model of DMD (Long et al., 2016; Nelson et al., 2016; Tabebordbar et al., 2016). In addition, Wei et al. (2016) provided proof‐of‐concept that CRISPR/Cas9‐mediated targeting of myostatin in skeletal muscle tissues prevents muscle wasting and partially preserves muscle function in a mouse model of cachexia. In these studies, Cas/sgRNA delivery into skeletal muscles was achieved via AAV infection, with the abovementioned problems.

The first successful applications of nanotechnology for gene editing strategies are now arising. Recent reports have shown the feasibility of using different types of nanostructures as nonviral vectors for the delivery of CRISPR components (e.g., Cas9 protein or mRNA, sgRNA, and DNA template) into several tissues and organs. For instance, lipid NPs have been shown to effectively deliver Cas9 mRNA and sgRNA to repair the *Angptl3* gene in the liver (Qiu et al., 2021), whereas gold NPs modified with polyethyleneimine (PEI) have been use to induce homology‐directed repair of hematopoietic stem and progenitor cells (Shahbazi et al., 2019). Recently, a biodegradable nanocapsule, has been used by G. Chen et al. (2019) to efficiently deliver a Cas9 ribonucleoprotein into different tissues and organs, including skeletal muscle. Finally, a collaborative effort between the Murthy and Conboy labs, allowed obtaining a fully repaired dystrophin protein through an HDR mechanisms via local injection of gold NPs containing Cas9/sgRNA RNP and donor DNA into dystrophic muscles **(**Figure 3 **)**. However, the efficiency of the process was low, likely due to the poor targeting of the satellite cell compartment (Lee et al., 2017). Therefore, strategies that efficiently target proliferating satellite cells, skeletal muscle fibers, and cardiac tissue emerge as a key issue for future nanotechnology‐based approaches for muscle diseases.

### 3.3 Nanosystems to modulate the immune response

As with other degenerative diseases, many MDs are associated with an aberrant immune response due to the degeneration‐induced necrosis and subsequent recruitment of the inflammatory infiltrate. Whereas under physiological circumstances, inflammation is a fundamental part of the pro‐regenerative response, needed for the activation and differentiation of muscle progenitors, in some MDs, such as DMD, the continuous release of the myofiber content upon degeneration leads to sustained inflammation, which contributes to the pathogenesis of the disease (Gallardo et al., 2021; Tidball, 2011). Therefore, it is not surprising that strategies aimed at modulating aberrant inflammation in dystrophic muscles are now in clinical practice. The standard‐of‐care treatment for DMD relies on corticosteroids, such as prednisone or deflazacort, to palliate the disease symptoms (Mercuri et al., 2019; Waldrop & Flanigan, 2019). Immunomodulation appears as a relevant therapeutic approach for other physio‐pathological skeletal muscle conditions, such as sarcopenia. During the aging process, there is a switch in the muscle microenvironment, which is usually associated with high levels of pro‐inflammatory cytokines, such as interleukin 6 (Li et al., 2020).

Despite their extended use in clinical practice, immunomodulatory agents have considerable adverse effects when administered at therapeutically relevant doses. Therefore, strategies aimed at reducing off‐target effects by lowering the effective dose or by increasing tissue and/ or target selectivity emerge as relevant clinical opportunities. Along these lines, some works have now started to use NPs as DDS to modulate the immune response in skeletal muscle. Conjugating the immunomodulatory agent to different types of nanostructures allows therapeutic levels of the drug while reducing toxic effects. For instance, PEG‐stabilized nanoliposomes were used to deliver the steroid pro‐drug methylprednisolone in a mouse model of DMD using (Turjeman et al., 2019). Treatment efficacy was shown by reduced inflammation and long‐term improvement of muscle strength.

**FIGURE 4:**
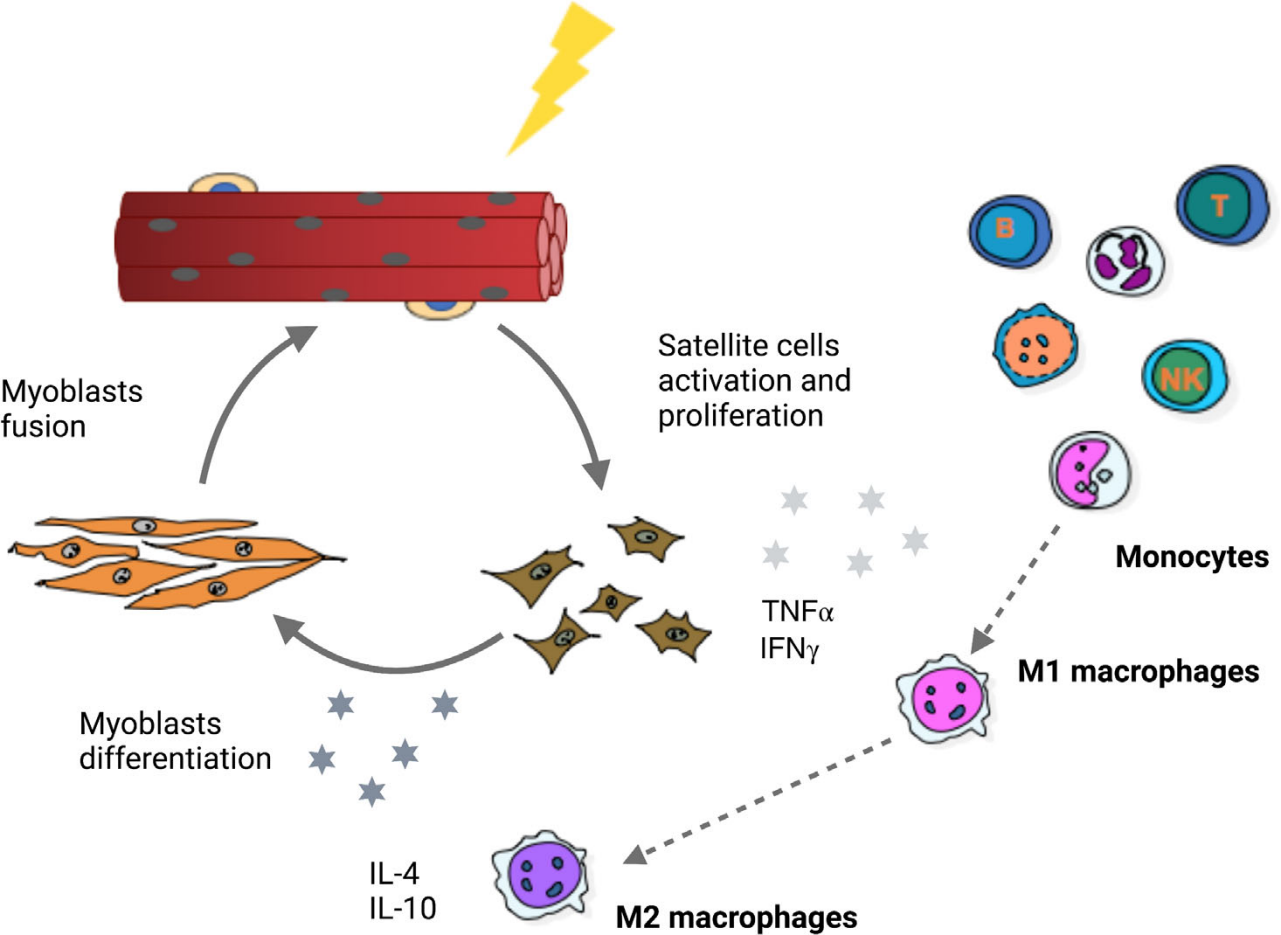
Immunomodulatory properties of inorganic nanoparticles (NPs) in skeletal muscle regeneration. Upon skeletal muscle injury, the recruitment of the immune system to the site of lesion is involved in the activation, proliferation and differentiation of a population of muscle‐resident stem cells (satellite cells). Monocytes differentiate into M1 macrophages, which regulate the activation and proliferation of satellite cells through the release of soluble cytoquines such as Tumor Necrosis Factor α (TNFα) and Inferferon γ (IFNγ). Upon phagocytosis of cellular debris, a switch of pro‐inflammatory M1 macrophages into anti‐inflammatory M2 macrophages mediates myoblast differentiation, allowing the completion of the regeneration program. Several types of inorganic NPs (gold, titanium oxide, and cerium oxide) induce M1‐to‐M2 macrophage polarization and could be used as immunomodulators in skeletal muscle disorders, characterized by aberrant inflammation

On the other hand, PEG‐stabilized gold NPs containing the macrophage polarizing cytokine IL‐4 have been shown to improve the muscle phenotype when delivered intra‐muscularly in a mouse model of ischemic injury (Raimondo & Mooney, 2018). Macrophages are the most abundant immune cells in regenerating muscles. Monocytes recruited to the site of lesion differentiate into pro‐inflammatory Ly6C+ M1 macrophages, which recruit other circulating monocytes, activate proliferation of muscle progenitors and are involved in the phagocytosis of cellular debris. Upon phagocytosis, M1 macrophages are switched into anti‐inflammatory Ly6C− M2 macrophages, which allows myogenic differentiation through the release of soluble factors (Arnold et al., 2007). Consistently, an imbalanced M1 to M2 polarization impairs skeletal muscle repair (Kharraz et al., 2013). Interestingly, recent works have shown that some inorganic NPs such as gold, titanium oxide and cerium oxide NPs may act as immunomodulators, through a mechanism involving macrophage polarization (Corsi et al., 2020). Although further studies are necessary to address the therapeutic relevance of these nanosystems, they may represent a promising tool for skeletal muscle disorders **(**Figure 4 **)**.

In addition to modulating the disease‐associated immune response, NP‐mediated drug delivery is showing tremendous promise in tampering AAV‐mediated immunogenicity in gene therapy applications. As discussed above, one of the main caveats of the current therapeutic approaches for monogenic diseases is the use of AAV vectors, which are highly immunogenic in humans (Duan, 2018). In a seminal study, Meliani et al. (2018) have recently demonstrated that rapamycin‐loaded PLA NPs inhibit the humoral response to AAV in mice and nonhuman primates, allowing vector redosing. This important observation suggests that combined treatments may help solve one of the major caveats of AAV‐delivered gene therapies.

### 3.4 Tissue targeting: Engineering the next generation of nanosystems for selective muscle delivery

When considering a nanotherapeutic approach for muscle disorders, the need to selectively treat such a vast tissue with limited off‐target effects poses an extra challenge. To date, all the clinically approved nanotherapies, excluding the two recently approved COVID‐19 mRNA vaccines from Pfizer/BioNTech and Moderna, are used as anti‐cancer treatments (e.g., Doxil, Onivyde, etc.). Cancer nanotherapies achieve tumor selectivity by exploiting the phenomena known as enhanced permeability and retention effect (Enhanced permeability and retention effect). This process increases the selective accumulation of NPs in the tumor area due to defects in the vasculature system generated during the tumor growth (Maeda, 2015). Studies in animal models had shown that the EPR effect could lead to a 50‐fold accumulation in tumors with respect to healthy tissues, although it provides only modest tumor specificity in humans (Cole & Holland, 2015).

However, for nontumor targets as skeletal muscle, there is the need to develop other strategies to increase the selectivity of the nanotherapeutics, and targeted delivery is emerging as the gold standard for future therapies. Several approaches are currently being explored, such as magnetic targeting. A magnetic NP is directed through the application of an electromagnetic field or active targeting, in which a targeting moiety on the surface of the nanostructure directs it towards a specific cell, tissue or organ. Several targeting moieties, such as antibodies, small molecules, and aptamers, interact with remarkable selectivity with receptors in the cell surface and can be conjugated to the NPs (Rothdiener et al., 2010; C.‐H. Wang, Kang, et al., 2012). For instance, and as mentioned above, different targeting peptides have been shown to efficiently target muscle cells in vitro and in vivo. In their work, Jativa et al. (2019) used G5‐PAMAM conjugated to the muscle homing heptapeptide ASSLNIA (Samoylova & Smith, 1999) to enhance gene delivery into C2C12 muscle cells. Similarly, Acharya and Hill (2014) used a cysteine terminated KDEL (Lys–Asp–Glu–Leu) peptide, to deliver gold NPs containing siRNAs into the same cell line.

Interestingly, selective muscle targeting has also been achieved in vivo in different mouse models. For instance, PLGA‐PEG NPs functionalized with a skeletal muscle targeting 12‐mer peptide (M12) (X. Gao et al., 2014) have been recently used to deliver PTEN inhibitors into dystrophic mdx mice (D. Huang et al., 2020). A different approach, based on the use of enzyme‐targeted NPs, was recently used to target peptide‐conjugated polymeric NPs into ischemic muscle via system delivery (Ungerleider et al., 2017). This approach exploits the abundance of ECM metalloproteinases in the remodeling muscle (Muhs et al., 2003), which induce selective MM9‐mediated peptide cleavage, and induction of a conformational change of the NP.

In addition to homing peptides, both small molecules and aptamers are currently explored as targeting molecules. Aptamers are a class of nucleic acid ligands, which are biocompatible, have low immunogenicity, small size, a high binding affinity to the target molecule, and are easy to modify (Zhou et al., 2016). Previous work has demonstrated that aptamer‐functionalized NPs can be used to selectively target cancer cells both in vitro and in vivo (Latorre et al., 2014). Recently, muscle‐specific aptamers have been developed using a cell internalization Systematic Evolution of Ligands by Exponential Enrichment (SELEX) approach (Philippou et al., 2018) and further studies will help to elucidate if they represent indeed an useful tool to increase NPs uptake into skeletal muscle fibers.

## 4 NANOMEDICINE: PITFALLS AND LIMITATIONS

Although nanotechnology‐based therapies promise new healthcare opportunities and represent a significant step towards personalized medicine, several issues and limitations need to be addressed and overcome. Bitter failures match the exciting discoveries obtained so far, such as the market approval of several nanodrugs (Colapicchioni, 2020).

The application of nanomedicine to muscle disorders is still in its infancy; however, the recent promising results in preclinical animal models are now opening the door to the possibility of clinical translation. At this stage, our experience with nanomedicine in other fields like cancer treatment or vaccine development will be fundamental. Even the most mature and successful applications of nanotechnology in medicine, the “cancer magic bullets,” have faced significant translational challenges. In numerical terms, the success rates for nanodrug potential candidates for Phases I, II, and III trials significantly plunge from 94% to 48% to 14%, respectively (Salvioni et al., 2019). This low product yield leads to a question: “is nanomedicine lost in translation”? When analyzing the factors that are limiting the efficient translation of nanomedicine from the bench to the bedside, the poor understanding of the interactions between NPs and the biological environment (i.e., body fluids, ECM, and cellular components) appears to be a crucial bottleneck (Hajipour et al., 2021). The NP physicochemical features are usually well characterized after their synthesis but they should also be systematically reevaluated in the context of their intended use (for instance in the biological medium where subsequent assays will be carried out) (Mahmoudi, 2021). As the FDA Nanotechnology Regulatory Science Research Plan state, “We intend our regulatory approach to be adaptive and flexible and to take into consideration the specific characteristics and the effects of nanomaterials in the particular biological context of each product and its intended use” (https://www.fda.gov/science-research/nanotechnology-programs-fda/fdas-approach-regulation-nanotechnology-products).

Once exposed to the bloodstream, NPs are immediately coated by a dynamic layer of proteins, lipids, metabolomes that is referred to as a “protein/ biomolecular corona.” By altering the synthetic identity of the NPs, this biomolecular corona significantly affects their pharmacokinetics, biodistribution and target capability and ultimately defines their fate in vivo (Mahmoudi, 2018; Salvati et al., 2013; Tekie et al., 2020). Although corrective efforts have been implemented by optimizing in vitro protocols mimicking in vivo conditions (Palchetti et al., 2016), several important factors including the significance of sex and age differences or health status, including comorbidities, have been overlooked (Hajipour et al., 2021). The importance of optimizing DDSs based on the pathophysiological characteristics has been recently discussed in closely related tissues, such as cardiac muscle (Lin et al., 2021). Given the similitudes between the two tissues, many of the key parameters discussed by Lin et al. (2021), such as the need of targeting the excessive immune response and the aberrant ECM deposition, will likely be fundamental also in skeletal muscle nanomedicine.

Finally, the contribution of sex will also need to be considered, as suggested from the recent analysis of the effectiveness and performance of the NP‐based vaccines against COVID‐19 developed by Pfizer/BioNTech and Moderna. Based on the available data, both vaccines showed a (slightly) better effectiveness in males than in females (Baden et al., 2021; Hajipour et al., 2021; Polack et al., 2020). It would be essential to pay more attention to these crucial biological and pathophysiological factors to accelerate a successful clinical translation of nanotherapeutics.

## 5 CONCLUSION

The clinical translation of nanotherapeutics is a long and complex process that requires addressing several issues, including biocompatibility and safety, interaction with the biological environment, efficiency, large‐scale production, cost, regulatory terms, and intellectual property (Hua et al., 2018). Nanotherapeutics and nanomaterials for skeletal muscle repair are still in their infancy, and several challenges and difficulties need to be considered before accessing to clinical trials. These include safety, poor understanding of in vivo behavior, reliability of animal models, and scaling‐up. (Zor et al., 2019). The fact that many promising solutions for skeletal muscle repair are based on inorganic materials poses additional concerns. Indeed, the in vivo translation of these nanomaterials is encountering great debates (Salvioni et al., 2019). Nowadays, only a few inorganic nanomaterials have been approved by the regulatory agencies, and a deeper understanding of their biological characteristics is required prior to clinical applications. Understanding the in vivo behavior of the different nanoplatforms for drug delivery is crucial to evaluate their effectiveness and biodegradability accurately. These critical issues are still scarcely investigated for nanotherapeutics applied to tissue regeneration.

On the other hand, biological barriers also influence the clinical translation of nanotherapeutics for promoting skeletal muscle regeneration. Among them, the vast size of the target tissue and the need to efficiently target also other organs such as heart. Similarly, the difficulty of reaching damaged muscle fibers needs to be carefully addressed in preclinical studies, as NPs may be retained at ECM, in particular in advanced phases of the disease. Finally, in the case of large physical injuries or when considering genetic neuromuscular diseases, a successful nanotherapeutic approach also needs to promote innervation to the newly regenerated muscle in order to recover a fully functional tissue.

Based on the lessons learned from cancer nanomedicine (only 14% of nanodrugs concluded Phase III with positive outcomes (Salvioni et al., 2019)) and from the last 40 years of Alzheimer's disease research (clinical failure rate of 99.6%; Mullane & Williams, 2019), the limited reliability of current animal models is a critical issue. The development of scalable manufacturing processes to good manufacturing practice (GMP) standards is another crucial issue to address. Nanocarriers' synthetic methods are generally mature and allow to produce NPs as highly reproducible populations (Anselmo & Mitragotri, 2019; Boselli et al., 2020). However, their complete characterization still needs standardization (Mahmoudi, 2021). While the regulatory agencies and entities such as European Nanomedicine Characterization Laboratory are actively working on a regulatory framework for nanomedicine, academic labs are still discussing how to implement a standardized reporting checklist for bio‐nanopapers (Minimum Information Reporting in Bio‐Nano Experimental Literature [MIRIBEL]) (Leong et al., 2019).

On the other hand, nanopatterned scaffolds incorporate a greater degree of complexity as compared to DDS, both in their synthesis and in their management by clinicians (Iravani & Varma, 2019). In this context, microfluidic‐based techniques, such as lithography or 3D bioprinting, are emerging as appealing candidates in mimicking skeletal muscle tissues in an automated and controlled manner, potentially facilitating a faster and smoother scale‐up of tissue constructs. (Jana et al., 2016; Ostrovidov et al., 2019; Zhuang et al., 2020). However, these technologies are still immature and further development is needed to overcome some technical challenges.

To conclude, nanotechnologies provide excellent candidate tools for precision and personalized medicine. Considering the complexity of the regenerative process, the possibility to optimize nanotherapeutics and nanopatterned scaffolds according to the genetic profile and responsiveness of the patient makes personalized nanomedicine the *Holy Grail* of tissue engineering. Nanotechnology is expected to provide unique features and new methodologies to control the regenerative process, by contributing to the development of smart, multi‐responsive materials with a controllable and robust delivery.

## CONFLICT OF INTEREST

The authors declare no potential conflict of interest.

## AUTHOR CONTRIBUTIONS

**Valentina Colapicchioni:** Conceptualization (equal); writing – original draft (equal); writing – review and editing (equal). **Francesco Millozzi:** Writing – original draft (supporting). **Ornella Parolini:** Writing – review and editing (supporting). **Daniela Palacios:** Conceptualization (equal); funding acquisition (lead); writing – original draft (equal); writing – review and editing (equal).

## RELATED WIREs ARTICLE

Nanotherapy for Duchenne Muscular Dystrophy

## Data Availability

Data sharing is not applicable to this article as no new data were created or analyzed in this study
